# Determinants of End-of-Life Expenditures in Patients with Oral Cancer in Taiwan: A Population-Based Study

**DOI:** 10.1371/journal.pone.0126482

**Published:** 2015-05-06

**Authors:** Ching-Chih Lee, Ting-Shou Chang, Cheng-Jung Wu, Ching-Chieh Yang, Po-Chun Chen

**Affiliations:** 1 Department of Otolaryngology, Dalin Tzu Chi Hospital, Buddhist Tzu Chi Medical Foundation, Chiayi, Taiwan; 2 School of Medicine, Tzu Chi University, Hualian, Taiwan; 3 Department of Otolaryngology, Kaohsiung Veterans General Hospital, Kaohsiung, Taiwan; 4 Department of Otolaryngology, Shung Ho Hospital, Taipei Medical University, Taipei, Taiwan; 5 Department of Radiation Oncology, Chi-Mei Medical Center, Tainan, Taiwan; 6 Department of Radiation Oncology, Pingtung Christian Hospital, Pingtung, Taiwan; University Medical Center of Princeton/Rutgers Robert Wood Johnson Medical School, UNITED STATES

## Abstract

**Background:**

To investigate the association of basic demographic data, socioeconomic status, medical services, and hospital characteristics with end-of-life expenditure in patients with oral cancer in Taiwan who died between 2009 to 2011.

**Methods:**

This nationwide population-based, retrospective cohort study identified 5,386 patients who died from oral cancer. We evaluated medical cost in the last month of life by universal health insurance. The impact of each variable on the end-of-life expenditure was examined by hierarchical generalized linear model (HGLM) using a hospital-level random-intercept model.

**Results:**

The mean medical cost in the last six months of life was $2,611±3,329 (U.S. dollars). In HGLM using a random-intercept model, we found that patients younger than 65 years had an additional cost of $819 over those aged ≥65 years. Patients who had a high Charlson Comorbidity Index Score (CCIS) had an additional $616 cost over those with a low CCIS. Those who survived post-diagnosis less than 6 months had an additional $659 in expenses over those who survived more than 24 months. Medical cost was $249 more for patients who had medium to high individual SES, and $319 more for those who were treated by non-oncologists.

**Conclusion:**

This study provides useful information for decision makers in understanding end-of-life expenditure in oral cancer. We found significantly increased end-of-life expenditure in patients if they were younger than 65 years or treated by non-oncologists, or had high CCIS, medium to high individual SES, and survival of less than 6 months after diagnosis.

## Introduction

Oral cancer is common worldwide, with an estimated 263,900 new cases in 2008 [1]. The current treatment is either surgery alone or in combination with radiotherapy and chemotherapy. However, the economic cost of advanced head and neck cancer is substantial and higher than that of other solid tumors [2–4]. Oral cancer has been ranked fourth in incidence and mortality in Taiwanese males since 1995. The estimated cost of treatment was approximately $1.195 billion U.S. dollars in Taiwan in 2004.

Previous studies have reported increasing aggressiveness in treatment (e.g., chemotherapy, ICU utilization, emergency department visits) for cancer near the end of life, especially in patients who received primary care from oncologists, those with higher individual socioeconomic status (SES), males and those with shorter post-diagnosis survival [5, 6]. However, the association between end-of-life expenditure and these parameters in oral cancer require further investigation. At present, no publication has shown which factors influence end-of-life expenditute in oral cancer. We hope to provide information on medical cost to help decision makers characterize the end-of-life expenditure associated with oral cancer.

This study uses the nationwide claims data from Taiwan’s National Health Insurance Research Database (NHIRD) for patients who died from oral cancer between 2009 and 2011 to investigate the determinants of end-of-life expenditure for those with oral cancer in the last month of life. This database provides basic demographic data as well as socioeconomic status, medical services, and hospital characteristics.

## Patients and Methods

### Ethical consideration

This study was approved by the Institutional Review Board of Buddhist Dalin Tzu Chi General Hospital, Taiwan. Review board requirements for written informed consent were waived because all personal identifying information had been removed from the dataset prior to analysis.

### Study design and data sources

We identified patients who died from oral cancer from Taiwan’s NHIRD and Taiwan’s National Health Insurance (NHI) Program between 2009 and 2011 [7]. Taiwan’s NHIRD captures basic demographic data, SES, medical services and hospital characteristics. The NHI Program contains information on billing and provides universal health insurance coverage for a comprehensive array of medical services for all of Taiwan’s residents. We excluded patients younger than 18 years of age in order to provide a more precise age distribution. There were only 2 patients younger than 18 years of age each year in this study. The definition of end-of-life expenditures in our study was the medical cost in the last month of life. It was observed that the cost of every medical measurement has experienced little changes throughout these years in Taiwan. The exchange rate is 30 Taiwan dollars to 1 U.S. dollar in this study, which is similar to current exchange rate.

### Measurement

#### Patient demographics

Patient characteristics included age, gender, post-diagnosis survival time, geographic location, urbanization level of residence, individual SES, status of advanced cancer and severity of comorbidity. The recoding and definition of SES and urbanization of residence in the insurance premium is based on income in Taiwan and several urbanization variables [8]. Severity of comorbidity was based on the modified Charlson Comorbidity Index Score (CCIS) recorded on the claims database for the last six months of each patient’s life. The CCIS is a widely accepted scale used for risk adjustment in administrative claims data sets [9]. Oral cancer metastatic status was identified by International Classification of Diseases, 9^th^ Revision (ICD-9) codes 196.xx to 199.xx.

#### Characteristics of physicians and hospitals

The primary physician’s specialty was identified from NHI claims and was dichotomized into oncologist vs. other. Hospitals were categorized by hospital level (medical center, regional or district hospital), caseload volume (high, medium or low) and hospital spending intensity (high or low). The hospital caseload volume was sorted by total patient volume using unique hospital identifiers [10]. To simplify the presentation, we defined the hospital volume into three categories: low, medium, and high. The volume category cutoff points were determined by sorting samples into three approximately equal groups.

### Statistical analysis

The key dependent variable of interest was medical expenditure in the last month of life. This amount included charges paid by both patients and the NHI Bureau in Taiwan. All statistical operations were performed using SPSS (version 15, SPSS Inc., Chicago, IL). The goodness of fit between the association of the end-of-life expenditures and explanatory variables by multiple linear regression was assessed with residual analysis, and we found that it was not suitable for implementing multiple linear regression due to unequal variance assumption (S1 Fig). Considering the clustering effect of physician compensation, procedures and policy in each hospital (S2 Fig), the impact of each variable on medical cost was examined by hierarchical generalized linear model using a hospital-level random-intercept model [11–13]. A two-tailed value of p<0.05 was considered significant.

## Results

A total of 5,386 patients who died from oral cancer from 2009 to 2011 were identified in the Taiwan NHIRD. Table 1 summarizes basic demographic data, socioeconomic status, medical services, hospital characteristics, and the end-of-life expenditures for patients with oral cancer. The mean age at death was 56±13 years. Most patients (72.5%) died between 35 and 64 years. In all, 3,445 patients (64%) presented with advanced disease (nodal metastasis or distant metastasis). Only 554 patients (10.3%) survived more than two years. Most patients (71.1%) were treated at a medical center.

**Table 1:** Baseline characteristics of the patient population.

Parameter			Year					
	Total		2009		2010		2011	
	No.	%	No.	%	No.	%	No.	%
Total	5,386	100	1,369	100	1,808	100	2,209	100
**Socioeconomic status**								
High	1,418	26.3	355	25.9	448	24.8	615	27.8
Medium	2,244	41.7	551	40.2	761	42.1	932	42.2
Low	1,724	32.0	463	33.8	599	33.1	662	30.0
**Gender**								
Male	5,033	93.4	1,281	93.6	1,670	92.4	2,082	94.3
Female	353	6.6	88	6.4	138	7.6	127	5.7
**Mean age, years** (±SD*)	56±13		56±13		56±13		57±12	
**Age group, years**								
18–34.99	99	1.8	36	2.6	32	1.8	31	1.4
35–64.99	3,906	72.5	987	72.1	1,313	72.6	1,606	72.7
more than 65	1,381	25.6	346	25.3	463	25.6	572	25.9
**CCIS** **								
0 or 1	1,907	35.4	479	35.0	665	36.8	763	34.5
2	1,038	19.3	283	20.7	333	18.4	422	19.1
3	398	7.4	102	7.5	135	7.5	161	7.3
4	2,043	37.9	505	36.9	675	37.3	863	39.1
**Cancer group**								
Local disease	1,941	36.0	499	36.4	662	36.6	780	35.3
Advanced disease	3,445	64.0	870	63.6	1,146	63.4	1,429	64.7
**Post-diagnosis survival, months**						
0–6	1,499	27.8	447	32.7	504	27.9	548	24.8
6.01–12	1,683	31.2	567	41.4	544	30.1	572	25.9
12.01–24	1,650	30.6	355	25.9	602	33.3	693	31.4
>24	554	10.3	0	0.0	158	8.7	396	17.9
**Primary physician’s specialty**								
Oncologist	831	15.4	213	15.6	284	15.7	334	15.1
Other	4,555	84.6	1,156	84.4	1,524	84.3	1,875	84.9
**Hospital characteristics**								
Medical center	3,238	71.1	975	71.2	1,291	71.4	1,562	70.7
Regional	1,461	27.1	364	26.6	486	26.9	611	27.7
District	97	1.8	30	2.2	31	1.7	36	1.6
**Caseload group**								
High	1,999	37.1	525	38.3	666	36.8	808	36.6
Medium	2,132	39.6	566	41.3	719	39.8	847	38.3
Low	1,255	23.3	278	20.3	423	23.4	554	25.1
**Urbanization**								
Urban	1,244	23.1	312	22.8	406	22.5	526	23.8
Suburban	2,361	43.8	614	44.9	787	43.5	960	43.5
Rural	1,781	33.1	443	32.4	615	34.0	723	32.7
**Geographic Region**								
Northern	2,351	43.7	609	44.5	767	42.5	975	44.2
Central	1,042	19.4	274	20.0	356	19.7	412	18.7
Southern	1,698	31.5	419	30.6	571	31.6	708	32.1
Eastern	291	5.4	67	4.9	111	6.1	113	5.1

*SD: standard deviation.

**CCIS: Charlson Comorbidity Index Score.

**Fig 1:**
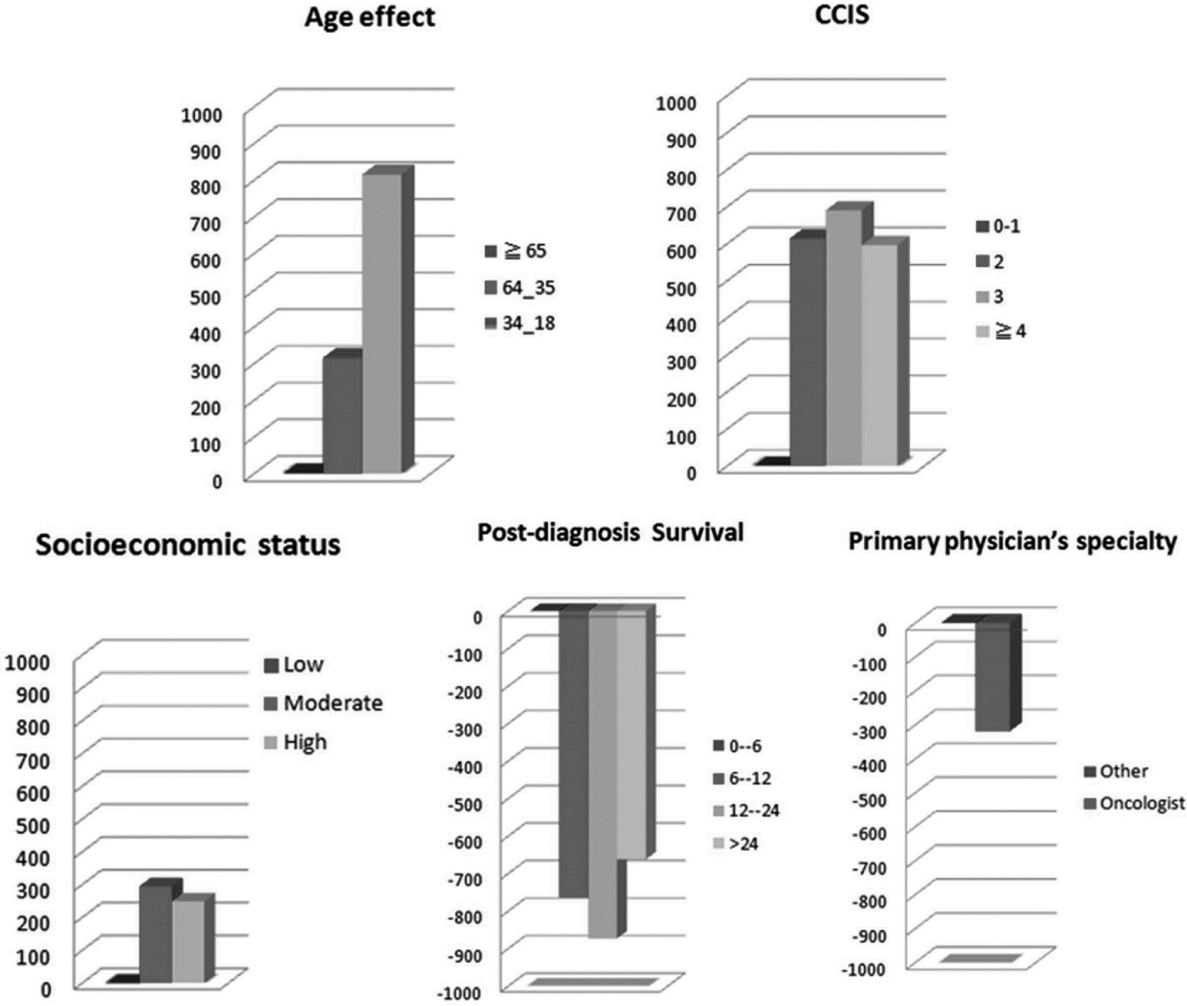
Factors associated with increased or decreased EOL expenditure in oral cancer decedents.

Fig 1 shows that younger age, increased CCIS, higher SES, shorter post-diagnosis survival, or treatment by the non-oncologists were associated with higher end-of-life expenditure. Patients younger than 35 years had the most substantial medical cost. In HGLM using a random-intercept model, we found several correlations of higher cost, listed in Table 2. The mean medical cost in the last month of life was $2,611 ± 3,329 (U.S. dollars). Patients younger than 35 years and those aged 35–64.99 years had a additional cost of $819 and $316, respectively, over those aged ≥65 years (*p<*0.001). Patients who had a CCIS of 2, 3 and 4 had an additional cost of $616, $692 and $597, respectively, over those with lower CCIS **(** *p<*0.001). Compared with those with post-diagnosis survival shorter than 6 months, those with longer post-diagnosis surival incurred less expense.

**Table 2:** Medical cost in the last one month of life of Taiwanese oral cancer decedents from 2009 to 2011 by hierarchical generalized linear model using a random-intercept model.

Parameter	Estimated cost (U.S. dollars)	P value
Intercept	2,470	<0.001
**Socioeconomic status**		
Low	Reference	
Moderate	292	0.007
High	249	0.037
**Gender**		
Male	Reference	
Female	-292	0.117
**Age group, years**		
More than 65	Reference	
35–64.99	316	0.004
18–34.99	819	0.018
**Charlson Comorbidity Index Score**		
0 or 1	Reference	
2	616	<0.001
3	692	<0.001
> = 4	597	<0.001
**Cancer group**		
Local disease	Reference	
Advanced disease	-2	0.984
**Post-diagnosis survival, months**		
0–6	Reference	
6.01–12	-761	<0.001
12.01–24	-871	<0.001
>24	-659	<0.001
**Primary physician’s specialty**		
Other	Reference	
Oncologist	-319	0.014
**Hospital characteristics**		
Medical center	Reference	
Regional	-31	0.848
District	-656	0.085
**Caseload group**		
High	Reference	
Medium	-92	0.549
Low	-217	0.292
**Urbanization**		
Urban	Reference	
Suburban	-197	0.116
Rural	-252	0.086
**Geographic Region**		
Northern	Reference	
Central	230	0.129
Southern	181	0.168
Eastern	126	0.603
**Year**		
2009	Reference	
2010	294	0.013
2011	75	0.520

*Medical cost of aggressive care in the last one month of life US dollars 2,611±3,329.

**95% CI, 95% confidence interval.

Patients with a medium or high individual SES had an additional cost of $292 and $249, versus to low individual SES (*p* = 0.007 and *p* = 0.037, respectively). Medical cost was $319 more in those who treated by the non-oncologists (*p* = 0.014).

## Discussion

From 2009 to 2011, our study identified 3,887 patients with oral cancer in Taiwan. We found significantly increased end-of-life expenditure in patients younger than 65 years of age, those with high CCIS, those treated by the non-oncologists, those of medium to high individual SES and those who survived less than 6 months after diagnosis.

The major strengths of this study are its characteristic as a nationwide population-based retrospective cohort study, with complete follow-up information captured by Taiwan’s NHIRD and NHI. This scheme provides comprehensive health care benefits with a moderate cost sharing and uses a uniform fee schedule to control health care costs. In 1995 it covered 92% of the population and had covered 99% by 2003. Medical cost is a reliable parameter of care because of the universal health insurance in Taiwan [14]. Hospital billing does not vary widely from one hospital to another. Furthermore, we assessed the impact of different variables on the medical expenditure with a hierarchical generalized linear model. There were obvious violations of the linearity and equal-variance assumptions in multiple linear regression. Significant clustering effect of medical expenditure was observed in hospital levels in our series. Due to above mentioned features in dataset, hierarchical generalized linear model, which extended generalized linear model with a hospital-level random intercept, was suggested [15,16]. Without using multi-level analysis and considering group-level variables, psychologistic fallacy which assumed the individual-level outcomes that could be totally explained by the individual-level variables may develop and the impact of main variables may be diluted [12].

Older patients receive much less aggressive end-of-life care. A meta-analysis [17] reported that non-treatment decisions occur more frequently in patients older than 80 years. Most publications also report less aggressiveness of end-of life care in the elderly [5, 6, 18]. In our study, patients varied greatly in medical cost by age. Medical cost was higher in patients younger than 65 years, especially those younger than 35 years. A possible explanation is that younger patients receive more aggressive end-of-life care. It is worth to investigate predictors for this group in the future.

Others have reported a trend of aggressiveness of end-of life cancer care for patients who were primarily cared for by an oncologist [19]. Patients cared for by oncologists were more likely to receive chemotherapy. However, we found higher medical cost in patients cared for by the non-oncologists. This discrepancy in results may be due to differences in the delivery of medical service among countries. In Taiwan, any practice of doctors can order administration of chemotherapy and hospitalize the patient.

Patients who had high levels of CCIS had higher medical cost than those with low levels of CCIS. Higher comorbidity scores were associated with higher rates of treatment complications and lower survival rates [20–22]. Nao et al. [23] found that high levels of comorbidity had a significant impact on the general complications rate after major surgery. In a study of patient group, Fiorentino et al. [24] found that acute toxicity after radiotherapy was mild and not influenced by the comorbidity score. Singh et al. [22] suggested that the presence of advanced comorbid conditions was not associated with an increase in the rate of treatment-associated complications, but comorbid condition might be more severe in such patients. Moreover, Lang et al. [4] found that patients diagnosed with advanced squamous cell carcinoma of the head and neck (SCCHN) had shorter survival time and higher medical cost than those with less advanced SCCHN. Clearly, there is an obvious correlation among shorter survival time, advanced disease, and higher medical cost. However, we did not find a significant association between advanced disease and higher medical cost. Contrary, we found that patients who survived less than 6 months post-diagnosis had higher medical cost than those who survived longer. Theoretically, a more severe disease usually requires more intense care, indirectly resulting higher expense. Perhaps, being aware of not living long due to serious diseases, also with the increasing popularity of hospice care, doctors and patients choose to have hospice care over intense care. Besides and in general, shorter post-diagnosis survival is more unlikely to be predicted which results the lack of intervention for the help of hospice care, and this might be the cause of high medical expense. We provide this information for doctors or patients to understand end-of-life expenditures in the years following their diagnosis, especially in the countries without universal health coverage. Although we can’t find out the predictors associated with survival in this study, for patients concerned about medical expenditures or who have longer life, their end-of-life expenditures will have no increase when compared to that of patients who have shorter life.

In our study, after controlling for other variables, low SES patients had lower medical costs, a result consistent with previous studies. Al-Refaie et al. [25] examined the impact of sociodemographic factors on receipt of complex cancer surgery at low-volume hospitals. Patients without private insurance were more likely to receive their cancer surgery at low-volume hospitals. Kong et al. [26] found that patients with lower household income or with residence in a rural location were more likely to be treated at low-volume hospitals. In such cases, patients with low SES have less access to medical resources. However, theoretically, in the system of universal health coverage, the levels of SES should not be the reason in affecting the use of medical resources. It appears that unknown reason exists. Perhaps, we could employ some approaches to enhance the use of medical resources in patients who have low SES, such as to advance accessibility, to reinforce the managerial skill in cases, and to invigorate the communication channel with patients. Nevertheless, our study meant to remind people that patients like this kind should receive special attention and care.

Our study has several limitations. First, the diagnosis of oral cancer and record of comorbid conditions are dependent on ICD codes. However, the NHI program in Taiwan reviews charts and interviews patients to verify the accuracy of diagnosis and treatment coding. The diagnosis of oral cancer was further verified by its inclusion in the catastrophic illness file. Second, we chose hierachical linear regression to analyze our data due to the clustering effect of the end-of-life expenditures among the hospital level. The interquartile range for median end-of-life expenditures was $ 1,132 ($923–2056) between different hospitals, and it represented the clustering effect of hospitals (S2 Fig). Third, medical expenditures resulting from malpractice or overtreatment were deducted. Although the “true” expenditure might not be obtainable from the present database, the results provide reasonably reliable information of the medical cost of this condition, according to the general standard of medical care after review of the NHI Program.

In the modern era, decision makers must not only provide optimal medical treatment but also consider the cost of futile medical care. Decision makers always follow the principle of providing appropriate cancer treatment. However, it can be difficult for them to determine when to begin hospice care. In this study, we provide decision makers information on medical cost for end-of-life expenditures in patients with oral cancer.

## Conclusion

We found significantly increased end-of-life expenditure in patients who were aged younger than 65 years, those with high CCIS, those treated by the non-oncologists, those with medium to high levels of individual SES and those who survived less than 6 months after diagnosis. These parameters may help healthcare providers and those responsible for health policy better understand this patient population so as to make informed decisions on how to reduce healthcare expenditures in the future.

## Supporting Information

S1 Fig The residual plot showed the standardized residual was not compatible with linear regression (residual >3). (TIF)

S2 Fig The box-plot for the EOL expenditure among oral cancer patients in different hospitals. (TIF)
