# Supplementary figures and images for: Determinants of End-of-Life Expenditures in Patients with Oral Cancer in Taiwan: A Population-Based Study
Source: PLoS One. 2015 May 6;10(5):e0126482. doi: 10.1371/journal.pone.0126482 (PMC4422718; doi:10.1371/journal.pone.0126482)

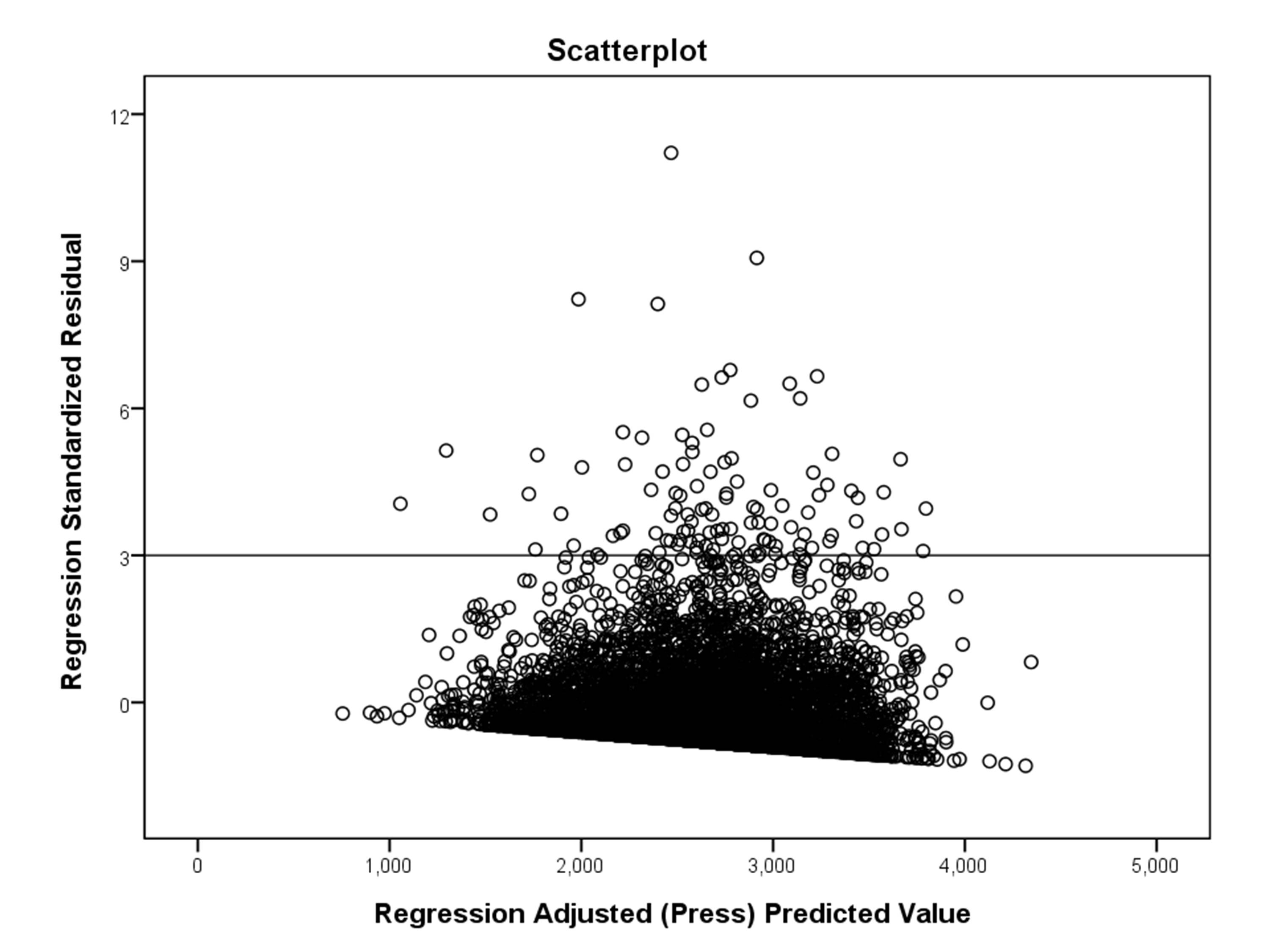

Supplement: S1 Fig — (TIF) [file pone.0126482.s001.tif]

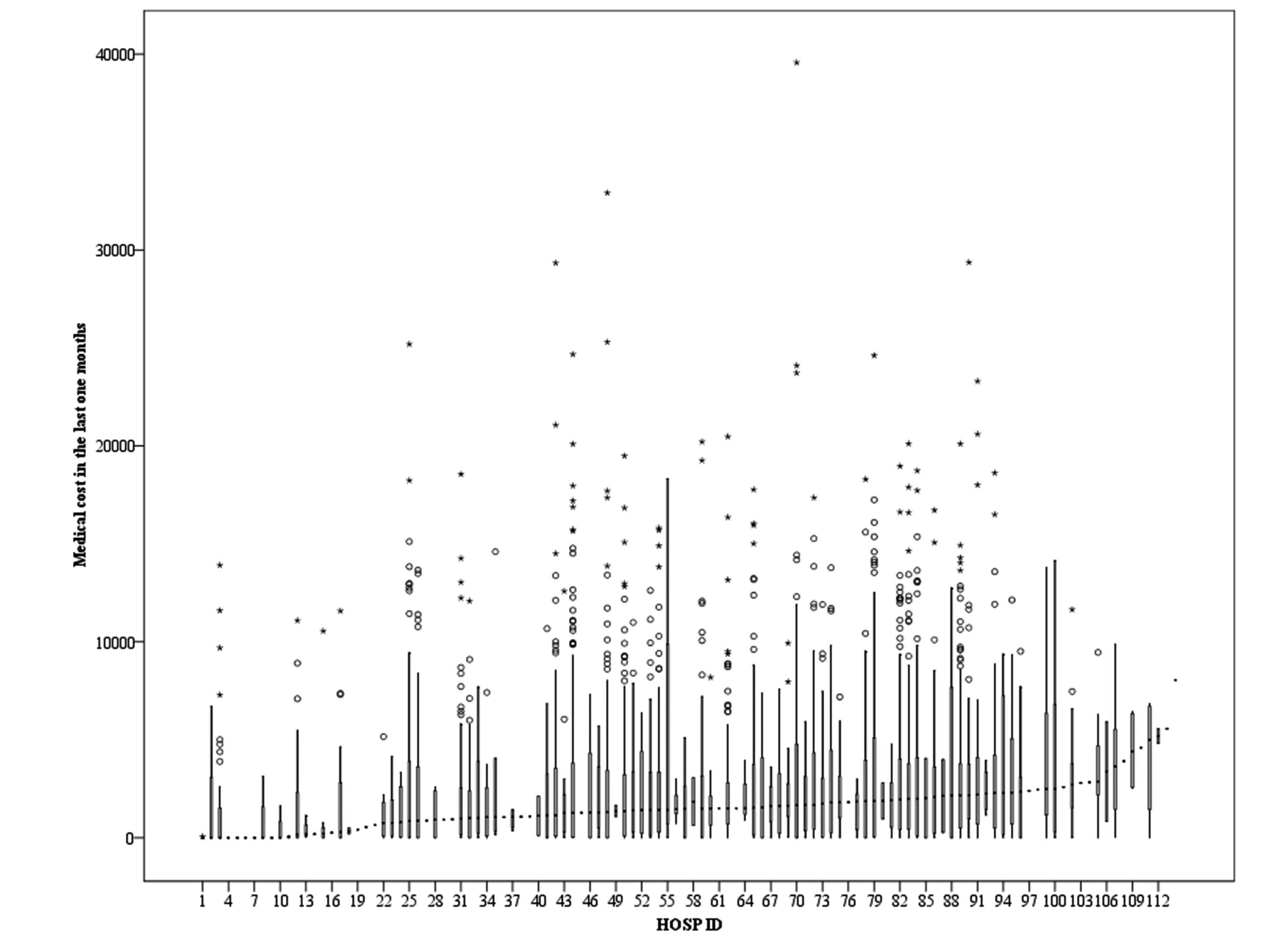

Supplement: S2 Fig — (TIF) [file pone.0126482.s002.tif]
